# High-Intensity Interval Training for Rowing: Acute Responses in National-Level Adolescent Males

**DOI:** 10.3390/ijerph19138132

**Published:** 2022-07-02

**Authors:** Emanuela Faelli, Marco Panascì, Vittoria Ferrando, Roberto Codella, Ambra Bisio, Piero Ruggeri

**Affiliations:** 1 Department of Experimental Medicine, Section of Human Physiology, University of Genoa, 16132 Genoa, Italy; emanuela.faelli@unige.it (E.F.); marco.panasci87@gmail.com (M.P.); ambra.bisio@unige.it (A.B.); ruggeri@unige.it (P.R.); 2 Centro Polifunzionale di Scienze Motorie, University of Genoa, 16132 Genoa, Italy; 3 Department of Biomedical Sciences for Health, Università degli Studi di Milano, 20133 Milan, Italy; roberto.codella@unimi.it; 4 Department of Endocrinology, Nutrition and Metabolic Diseases, IRCCS MultiMedica, 20900 Milan, Italy

**Keywords:** HIIT, rowing, workload, adolescent, RPE

## Abstract

Background: This study investigated the acute effects of two high-intensity interval training (HIIT) programs on physiological responses and internal workload. Methods: Ten national-level adolescent male rowers (age: 15.7 ± 0.2 years; maximal oxygen uptake (VO_2_max): 60.11 ± 1.91 mL∙kg^−1^∙min^−1^) performed two HIIT testing sessions: short (S-HIIT) and long (L-HIIT). In S-HIIT, the rowers performed 25 reps of 30 s at 100% power at VO_2_max (Pmax) interspersed with 30 s at P@20% Pmax; whereas in L-HIIT, the rowers executed 4 × 4 min at P@90% Pmax interspersed with 3 min of active recovery (P@30% Pmax). Results: The acute physiological responses and internal workload were evaluated. The significance level was set at *p* < 0.05. Oxygen uptake (VO_2_) (*p* < 0.05), time spent per session at ~90% VO_2_max (*p* < 0.01), total VO_2_ consumed (*p* < 0.01), total distance (*p* < 0.001), the rating of perceived exertion, blood lactate concentration and heart rate (always *p* < 0.0001) were significantly higher in L-HIIT than in S-HIIT. However, peak power output was significantly lower in L-HIIT compared to S-HIIT (*p* < 0.0001). Conclusion: In adolescent rowers, both HIIT tests stimulated aerobic and anaerobic systems. The L-HIIT test was associated with acute cardiorespiratory and metabolic responses, as well as higher perceptions of effort than the S-HIIT test. In adolescent rowers, HIIT emerges as an asset and could be introduced into a traditional in-season, moderate-intensity and endurance-based rowing program once a week.

## 1. Introduction

Rowing is a sport with high demands on various components of physical fitness (such as endurance and strength) and physiological characteristics, which reach the highest levels in rowers that have been recorded for any sportspeople [1].

Typically, rowers perform large training volumes to develop correct technique and reach high physical capacities. Aerobic training is generally adopted for well-trained rowers who perform most of their training sessions at intensities below the first lactate threshold (LT), despite competing at much higher intensities [2]. However, additional increases in aerobic training may not result in improved physiological variables and endurance performance [3]. While continuous training relies primarily on the duration of the effort, high-intensity interval training (HIIT) repeatedly stresses physiological parameters above the actual requirements [4], thus eliciting significant increases in performance and fitness [5,6].

While continuous exercise only comprises workload intensity and total duration, intermittent exercise consists of several components, such as peak workload intensity, peak workload duration, recovery load and recovery duration, as well as mean load [7]. The number of intervals is an adjunct variable in the HIIT prescription.

The manipulation of training intensity/duration and recovery periods during HIIT leads to differentiated training adaptations [8], becoming determinant for training program design.

Previous studies on elite rowers have shown that intermittent exercise (HIIT) induces greater improvements in VO_2_max and power output at LT compared to continuous exercise [9,10]. Incorporating HIIT along with constant load exercise, such as moderate-intensity exercise (MICE), could thus enhance performance by a larger extent [11]. Generally, long-interval HIIT lasts for 2–8 min with work intensities at 85–100% of VO_2_max, using work:rest ratios of between 1:0.5 and 1:2. At these intensities, the total duration is typically 20–40 min, split into 3–8 repetitions. In contrast, short-interval HIIT durations are usually between 30 and 60 s with work:rest ratios of between 1:0.5 and 1:1. Therefore, for these interval sets, the total work duration is generally 10–20 min, split into 1–5 repetitions [12].

The studies mentioned above focused on adult athletes; however, adolescent rowers have unique profiles that must be cautiously considered when selecting training protocols. In fact, during exercise, the energy system generally hinges on oxidative pathways more in adolescents than in adult subjects [13]. However, other studies have pinpointed the contribution of anaerobic energy pathways in national-level adolescent rowers [14]. Indeed, Italian coaches generally use a polarized model that is very similar to the training regimes that are adopted internationally to plan the intensity and volume of training cycles, according to the Italian Rowing Federation’s guidelines (FIC). This model is characterized by ~80% of the total training volume being at an intensity below the first LT and another 15–20% of the training being at an intensity between the first and second LT. Finally, a very small volume (~2–5%) is performed above the second LT [12]. In summary, when all pathways are maximized, high levels of performance may be achieved.

Therefore, it is of paramount interest to evaluate the acute effects of different HIIT protocols on adolescent rowers, thereby providing sport scientists, coaches and athletes with new information and possibilities for personalizing training schedules.

The purpose of this study was to determine the acute cardiorespiratory and metabolic responses and internal workload that were produced by two different HIIT regimes, which were matched for exercise duration. We hypothesized that both tests would elicit aerobic and anaerobic responses.

## 2. Materials and Methods

### 2.1. Subjects

Ten national-level adolescent male rowers with at least (a) 3 years of experience in national competition settings and (b) a training volume of six sessions/week (90 min/session) were recruited. All subjects competed and were finalists in the Italian National Rowing Championships in the year preceding the investigation and five of them were medalists in the men’s four discipline (M4-). According to McKay et al. [15], these athletes can be classified as highly trained/national level. For adolescent rowers, the race distance in the Italian National Rowing Championships is 2000 m. Before the experimental protocol, the anthropometric characteristics and body composition of the subjects were assessed. Height was measured with a stadiometer (Seca 217, Vogel & Halke, Hamburg, Germany) while body weight (kg), lean mass (kg), body mass index (BMI; kg∙m^−2^) and fat mass (%) were evaluated using bioelectrical impedance analysis (BIA; Tanita, BC-420 MA, Tanita, Tokyo, Japan) with a 50 kHz frequency measurement. The body composition measurements were performed in the standing position, barefoot with the legs and thighs not touching and the hands placed 15 cm away from the body laterally. Before the measurements, the skin and electrodes were precleared and dried. Participants were asked to abstain from large meals the evening before the test and on the day of the measurement and then they neither ate nor drank before the end of the procedure. The characteristics of the rowers at the baseline are presented in Table 1.

Muscle or joint injuries and any other contraindications within six months of the commencement of the study were chosen as exclusion criteria. All subjects were tested in the pre-season, four months before the Italian National Rowing Championships. The subjects were instructed to avoid any consumption of food in the 3 h prior to the testing session and to refrain from caffeine intake in the previous 24 h. They were also recommended not to change their dietary habits during the intervention period and not to exercise in the 24 h prior to each experimental session. Before the experimental protocol, the adolescents’ parents were fully informed about the study aims and procedures and they provided their written informed consent for their children to participate in the study. The experimental protocol conformed to the Code of Ethics of the World Medical Association (Declaration of Helsinki) and was approved by the Ethics Committee of the University of Genoa (protocol code: 246; date of approval: 7 July 2020).

### 2.2. Sample Size

An *a priori* estimation of the sample size was performed using VO_2_max as one of the primary outcome measures [16]. The sample size was estimated using the GPower software (3.1 software, Heinrich-Heine-Universität, Düsseldorf, Germany) by applying one-way ANOVA (F-test) with a significant level of 0.05 and a statistical power of 80% to an effect size (ES) of 1.1 [17]. This calculation generated a desired sample size of at least 10 participants. 

### 2.3. Experimental Design

**Figure 1:**
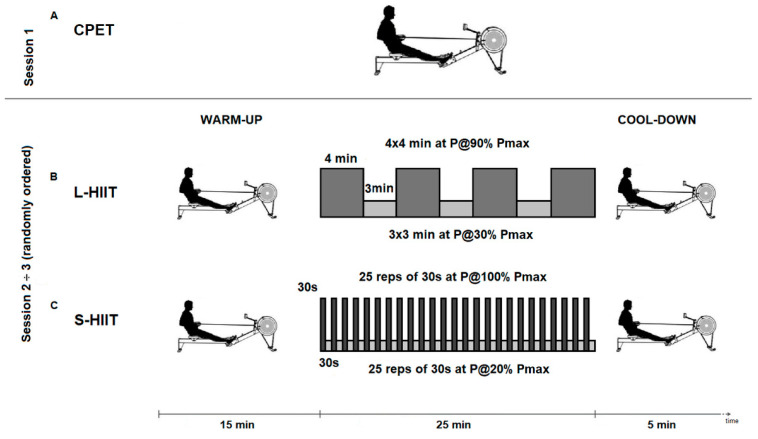
Experimental design: (**A**) the CPET session; (**B**) the L-HIIT testing session; (**C**) the S-HIIT testing session. CPET, cardiopulmonary exercise test; HIIT, high-intensity interval training; L-HIIT, long high-intensity interval training; S-HIIT, short high-intensity interval training; P, power; Pmax, power at VO_2_max.

A randomized crossover design consisting of three testing sessions that were separated by one week was carried out. The testing sessions involved a cardiopulmonary exercise test (CPET) and two HIIT sessions: L-HIIT (long) and S-HIIT (short). During the first session, the subjects underwent the CPET to determine their maximal oxygen uptake (VO_2_max) (mL·kg^−1^·min^−1^) and power at VO_2_max (Pmax) (Watt), which were used to quantify the intensity of the exercise during the HIIT sessions. Then, from the second to the third session, both HIIT regimens were completed in a randomized order. The randomization was conducted in draw form on the assessment day. Both testing sessions consisted of 15 min of warm-up, followed by 25 min of HIIT exercise plus 5 min of cool-down and lasted for 45 min (Figure 1).

#### 2.3.1. Cardiopulmonary Exercise Test (CPET)

The CPET was performed on a rowing ergometer (Concept 2, Model D, Morrisville, VT, USA) using an ergospirometer (Sensormedics, Viasys, Irvine, CA, USA) and a mask with a dead space of 30 mL (Hans Rudolph, INC., Shawnee, KS, USA). During the test, the expired gas was sampled, breath by breath. 

The CPET was performed on a Concept 2 ergometer. The drag factor settings were adjusted to 115, as recommended by the Italian Rowing Federation (FIC) for adolescent male rowers [18]. The participants completed a standardized 10 min warm-up, consisting of 4 min at 120 W, 3 min at 150 W and 3 min at 120 W; then, the incremental maximal test started with a 3-min rowing session at 150 W and was subsequently intensified in 25 W increments each minute [19]. The cardiorespiratory data were continuously recorded during the CPET, while the blood lactate concentration [La]^+^ and rating of perceived exertion (RPE) were measured 2 min after the end of the CPET.

Then, [La]^+^ was measured with fingertip blood samples (5 μL) using a Lactate Pro 2 (LP, Arkray KDK, Koka-shi, Shiga, Japan) [20], while the RPE was assessed using the Borg CR10 scale [21].

The VO_2_max of the rowers was considered to be reached when at least three of the following criteria were fulfilled: (i) a steady state of VO_2_, despite increasing power output (change in VO_2_ ≤ 150 mL·kg^−1^·min^−1^ at VO_2_max); (ii) a final respiratory exchange ratio (RER) that exceeded 1.1; (iii) visible exhaustion; (iv) an HR at the end of the exercise (HRmax) within 10 bpm of the predicted maximum (210 − (0.65 × age)); and (v) a lactate concentration at the end of the exercise ([La]^+^) that was higher than 9 mmol∙L^−1^ [22,23,24]. The lowest power needed to elicit the VO_2_max was defined as Pmax [8] and was considered as the power that corresponded to the last one-minute step that was completed during the CPET.

The characteristics of the participants after the CPET are reported in Table 2.

#### 2.3.2. HIIT Testing Sessions

The HIIT tests were performed on a Concept 2 ergometer with a drag factor of 115 [18] in a laboratory with a controlled temperature and humidity (21–24 °C and 44–56%, respectively) and at the same time of the day (11:00 a.m. ±1 h) to avoid any influence of circadian rhythms. The experimental protocol was generally well tolerated and the subjects completed the testing sessions without complications. The participants did not report any dizziness, lightheadedness or nausea symptoms. 

In both HIIT tests, the rowers performed 10 min at 40% of their HRmax plus 5 min at the athlete’s preferred pace as a warm-up and 5 min at P@10% Pmax as a cool-down (Figure 1). 

In the L-HIIT test, the rowers performed 4 × 4 min at P@90% Pmax (30 ÷ 32 str∙min^–1^) interspersed with 3 × 3 min of active recovery (P@30% Pmax) for a total accumulated duration of 25 min, while in the S-HIIT test, they performed 25 reps of 30 s at P@100% Pmax (34 ÷ 36 str∙min^–1^) interspersed with 30 s at P@20% Pmax for a total duration of 25 min (Figure 1). All testing sessions were supervised by an Italian Rowing Federation (FIC) certified trainer.

### 2.4. Outcome Measures

#### 2.4.1. Physiological Responses

Oxygen uptake (VO_2_), the volume of carbon dioxide exhaled (VCO_2_), respiratory exchange ratio (RER), time spent per session in exercise bouts at an intensity close to or above 90% VO_2_max (T@90% VO_2_max), total VO_2_ consumed (TotVO_2_) and ventilation (VE) were continuously monitored during the exercise. The average of VO_2_ value, which was obtained during the last 30 s of the final rowing stage, was considered as the VO_2_max while the RER was averaged over the last minute of each rowing step. The TotVO_2_ was calculated by the summation of each VO_2_ value (mL·kg^−1^), which were measured breath by breath throughout the whole exercise [25].

#### 2.4.2. Internal Workload

The RPE, [La]^+^ and HR were measured as indices of internal workload. The use of RPE has been demonstrated to be a simple and inexpensive tool to provide a quantification of internal workload [26] and can be related to physiological markers to understand the responses to a specific exercise intensity [27]. Two minutes after the end of each HIIT session, both the RPE [28] and [La]^+^ [29] were assessed.

The RPE was measured using the Borg CR10 scale [21], which is a category–ratio scale that ranges from 0 (no effort at all) to 10 (maximum effort ever experienced). Before the commencement of the study, the subjects were familiarized on both the use of the scale and the anchoring procedures. 

The [La]^+^ was assessed with fingertip blood samples (5 μL) using a Lactate Pro 2 (LP, Arkray KDK, Koka-shi, Shiga, Japan). Before each collection and according to the recommendations of the manufacturer, the finger was cleansed with alcohol and allowed to air dry [20].

HR was continuously monitored during the exercises and was recorded at 5-s intervals with a Polar heart rate monitor (Polar H7, Electro, Kempele, Finland). HRmax was identified as the highest value that was achieved during the exercise.

#### 2.4.3. Performance Parameters

The total distance completed (TD) and the peak power output (PPO) were assessed as indices of the rowing performance. TD was measured as the total distance that was covered by the rower during each testing session, while PPO was recorded as the highest power output (or peak value) that was achieved during each test [30].

### 2.5. Statistical Analysis

All data were normally distributed, according to the Shapiro–Wilk test, except for T@90% VO_2_max (raw values and percentages) and VE. We compared the effects of S-HIIT and L-HIIT on physiological measures (i.e., VO_2_, VCO_2_, T@90% VO_2_max, RER and TotVO_2_), performance parameters (TD and PPO) and internal workload (RPE, HR and [La]^+^). The normally distributed data (VO_2_, VCO_2_, RER, HRmax, [La]^+^, TotVO_2_, TD, PPO and RPE) were statistically evaluated by means of a paired t-test. The T@90% VO_2_max (raw data and expressed as a percentage of the total exercise duration) and VE values were analyzed by means of the Wilcoxon test. Cohen’s d was used to estimate the effect size, considering the cut-off points of 0.10, 0.25 and 0.40, which represented a small, medium and large effect, respectively [31]. The significance level was set at *p* < 0.05. The normally distributed data are provided as the mean ± SE; not normally distributed data are presented as the median (interquartile range). The 95% confidence interval (CI) is also reported.

## 3. Results

The mean (median) and standard error (interquartile range) values for both L-HIIT and S-HIIT and the results of the statistical analyses are reported in Table 3. 

### 3.1. Physiological Responses

The results of the statistical analysis showed that VO_2_ in S-HIIT was significantly lower than in L-HIIT. A significant effect of the session was shown by *t*-test on the TotVO_2_ values, revealing that TotVO_2_ in S-HIIT was significantly lower than in L-HIIT. Moreover, VCO_2_ values in S-HIIT were significantly lower than in L-HIIT. The Wilcoxon test showed that VE was significantly lower in S-HIIT with respect to L-HIIT, while the T@90% VO_2_max in L-HIIT was significantly longer than in S-HIIT. The strength of these results was supported by the large effect size values for all of the parameters. 

### 3.2. Internal Workload

As a result of the statistical analysis, the RPE, HR and [La]^+^ values were significantly higher in L-HIIT than in S-HIIT, with large effect size values. 

### 3.3. Performance Parameters

The *t*-test on the distance values showed that TD was significantly longer in L-HIIT than in S-HIIT, whereas PPO was significantly higher in S-HIIT than in L-HIIT. The statistical power was large, as shown by the effect size values. 

## 4. Discussion

In this study on national-level adolescent male rowers, we investigated the acute cardiorespiratory responses and internal workload following two high-intensity interval tests that were matched for exercise duration. 

The major finding was that the L-HIIT protocol stimulated both aerobic and anaerobic systems to a greater extent and with higher internal workload than the S-HIIT regime.

In rowing, energy supply is dependent on the functional capacity of oxidative and anaerobic pathways, with the relative contribution of the former being about 80%. Therefore, rowing highlights the challenge of simultaneously developing both endurance and muscle strength. Further, as a relatively higher energy cost is attributed to the drag that is created by wind and water resistance, rowers must ameliorate their technique to develop a more efficient recovery phase (particularly in the timing of the forces at the catch), as well as a faster stroke rate and a stronger propulsive stroke. Thus, the first challenge in a rower’s training, particularly for experienced athletes (including youth athletes), is to combine endurance and strength and, at the same time, optimize their technique. 

In youths, oxidative metabolism is appreciable during high-intensity exercise, which is likely associated with more efficient oxidative phosphorylation, oxygen delivery and utilization and/or muscle fiber recruitment patterns [32]. Further, in adolescents, anaerobic capacity is significantly related to the time spent at a high percentage of VO_2_max in high-intensity domains, which is different from adults [33]. 

In the present study, in order to assess the acute physiological responses of the aerobic energy system, we considered VO_2_ uptake, T@90% VO_2_max and TotVO_2_ as our primary variables of interest.

A comparison between the VO_2_ uptake and VO_2_max values that were measured during the CPET revealed that the VO_2_ value in L-HIIT (peak of 97% VO_2_max) was significantly higher (with a large effect size) than that measured in S-HIIT (VO_2_ uptake peaked at 90% VO_2_max), indicating that L-HIIT was more effective in attaining VO_2_max than S-HIIT. 

As the optimal stimulus for improving the cardiorespiratory function of athletes is maintaining long periods of time above 90% of their VO_2_max (i.e., in their “red zone”) [8], we used T@90% VO_2_max to compare the two HIIT regimes and identify the most effective protocol for stressing aerobic capacity. Under our experimental conditions, the T@90% VO_2_max was significantly longer in L-HIIT compared to S-HIIT.

In addition, the total oxygen consumption during exercise has been considered as a measure of aerobic energy yield [34]. In the present study, we found that the TotVO_2_ was significantly higher (with a large effect size) in L-HIIT than in S-HIIT.

On the whole, our study demonstrated the effectiveness of HIIT for stressing the aerobic systems of national-level adolescent male rowers, thereby supporting the notion that intermittent modality may induce significant improvements in cardiorespiratory adaptations in adolescents [35]. 

With regard to internal workload, the RPE that was measured at the end of both HIIT sessions significantly increased (with a large effect size) in L-HIIT with respect to S-HIIT. This result was also confirmed when matching the performed work to the perception of effort [36].

According to Buchheit and Laursen [8], L-HIIT and S-HIIT programs can be classified as very high (>14 mmol∙L^−1^) and moderate (>6 mmol∙L^−1^), respectively; thus, HIIT appears to be essential when the goal is to develop glycolytic pathways.

Our results showed that [La]^+^ was significantly higher (with a large effect size) in L-HIIT, indicating that long modality was an effective protocol for stressing both the aerobic and anaerobic energy systems. 

Interestingly, training above the LT not only improves lactate response and its adaptations, but these responses also do not compromise aerobic training adaptations [37]. Prescribing HIIT sessions could promote a delay in the accumulation of lactate, both by increasing the oxidative capacity and the recruitment of muscle fibers [38] and by ameliorating lactic tolerance [39]. However, our results should be cautiously scrutinized as all of the above-quoted studies were performed on moderate-endurance or well-trained adult athletes.

In addition, in accordance with a previous study [40], the comparison between the HR values and HRmax that were achieved in the CPET revealed that the HRmax in L-HIIT (99% HRmax) was significantly higher (with a large effect size) than in S-HIIT (95% HRmax). 

### Limitations

The absolute differences in the mean workloads rendered the comparability a limitation of this study. In addition, internal measures (RPE and [La]^+^) were only taken after the completion of the tests and not during the exercise, which was inherent to the experimental settings. Moreover, as VO_2_ was evaluated during the pre-season, this could represent a potential limitation for the sample size estimation, which could have further benefited from a larger sample size.

## 5. Conclusions

In the present study, we showed that for highly trained adolescent rowers, HIIT is a well-established and effective modality to stimulate aerobic and anaerobic systems. HIIT regimes have emerged as critically relevant, even within traditional moderate-intensity and endurance-based rowing programs. Considering our findings, L-HIIT could be introduced into a weekly training program once a week during the competitive season. Follow-up studies are needed to ascertain the extent to which these acute responses can be preserved in the long term, following L-HIIT and S-HIIT, for national-level adolescent rowers.

## Figures and Tables

**Table 1:** The characteristics of the rowers at the baseline. Data are the mean ± SE. BMI, body mass index.

Age (Years)	Height (cm)	Weight (kg)	BMI (kg∙m^−2^)	Fat Mass (%)	Lean Mass (kg)	Weekly Training Volume (h∙wk^−1^)	Rowing Experience (Years)
15.67 ± 0.22	179.78 ± 1.67	68.79 ± 2.58	21.22 ± 0.51	4.81 ± 0.79	60.63 ± 2.45	11.22 ± 0.26	4.11 ± 0.42

**Table 2:** The responses of the rowers after the cardiopulmonary exercise test (CPET). Data are the mean ± SE. VO_2_max, maximal oxygen uptake; Pmax, power at VO_2_max; [La]^+^, blood lactate concentration; RPE, rating of perceived exertion; HRmax, maximal heart rate.

VO_2_max (mL∙kg^−1^∙min^−1^)	Pmax (Watt)	[La]^+^ (mmol∙L^−1^)	RPE (AU)	HRmax (bpm)
60.11 ± 1.91	296.90 ± 12.45	18.44 ± 1.17	9.56 ± 0.23	198 ± 1.77

**Table 3:** The physiological responses, performance parameters and internal workload after long HIIT (L-HIIT) and short HIIT (S-HIIT). The normally distributed data are provided as the mean ± SE; not normally distributed data values are presented as the median (interquartile range). VO_2_, oxygen uptake; TotVO_2_, total oxygen uptake consumed; VCO_2_, volume of carbon dioxide exhaled; RER, respiratory exchange ratio; VE, ventilation; T@90% VO_2_max, time spent per session in exercise bouts at an intensity close to or above 90% of maximal oxygen uptake; TD, total distance; PPO, peak power output; RPE, rate of perceived exertion; HRmax, maximum heart rate; [La]^+^, blood lactate concentration, CI, confidence interval; d, Cohen’s d.

Variables	L-HIIT	S-HIIT	Statistical Analysis: L-HIIT vs. S-HIIT
*Physiological Responses*			
VO_2_ (mL∙kg^−1^∙min^−1^)	58.57 * (2.87) CI [52.08, 65.06]	52.50 (1.25) CI [49.84, 55.16]	t(9) = 2.35, *p* = 0.043, d = 0.74
VO_2_ (L∙min^−1^)	4.00 * (0.12) CI [3.73, 4.27]	3.62 (0.12) CI [3.35, 3.88]	t(9) = 2.52, *p* = 0.033, d = 0.80
TotVO_2_ (mL·kg^−1^)	1306.29 ** (70.45) CI [1146.91, 1465.67]	1131.65 (50.97) CI [1016.35, 1246.96]	t(9) = 4.47, *p* = 0.0016, d = 1.41
VCO_2_ (L∙min^−1^)	4.05 * (0.18) CI [3.64, 4.46]	3.47 (0.15) CI [13.15, 3.80]	t(9) = 2.68, *p* = 0.025, d = 0.85
RER	1.01 (0.03) CI [0.96, 1.08]	0.96 (0.02) CI [0.91, 1.00]	t(9) = 1.49, *p* = 0.17, d = 0.47
VE (L∙min^−1^)	115.75 * [105.90, 126.15] CI [84.33, 135.60]	109.95 [84.90, 116.40] CI [72.19, 119.92]	Z = −2.50, *p* = 0.013, d = 0.79
T@90% VO_2_max (s)	790.17 ** [678.50, 918.00] CI [709.13, 883.53]	493.22 [301.75, 738.50] CI [347.85, 643.03]	Z = −2.80, *p* = 0.005, d = 0.89
*Performance Parameters*			
TD (m)	5470.30 *** (159.70) CI [5109.04, 5831.56]	4863.60 (137.28) CI [4553.06, 5174.13]	t(9) = 6.50, *p* < 0.001, d = 2.06
PPO (W)	267.24 *** (9.98) CI [244.65, 289.82]	296.90 (11.03) CI [271.94, 321.85]	t(9) = 26.80, *p* < 0.0001, d = 9.11
*Internal Workload*			
RPE (A.U.)	9.22 ** [8.75, 10.00] CI [8.68, 9.81]	5.83 [5, 6] CI [5.19, 6.14]	Z = −2.82, *p* < 0.005, d = 0.89
HRmax (bpm)	196.60 *** (2.25) CI [191.51, 201.69]	188.80 (2.14) CI [183.96, 193.64]	t(9) = 8.17, *p* < 0.0001, d = 2.58
[La]^+^ (mmol∙L^−1^)	16.48 *** (1.2) CI [13.75, 19.21]	8.46 (1.05) CI [6.09, 10.82]	t(9) = 8.17, *p* < 0.0001, d = 2.54

* *p* < 0.05; ** *p* < 0.01; *** *p* < 0.001.

## Data Availability

The data presented in this study are available upon request.
